# Exploring the physiological limits of aging: a case study of the male 50-km world record in the 80+ age category

**DOI:** 10.3389/fphys.2025.1735019

**Published:** 2026-01-12

**Authors:** A. M. Pilotto, E. Higueras-Liébana, M. Ansaldo, I. Baltasar-Fernandez, M. Neri, L. Giusti, Á. Buendía-Romero, P. L. Valenzuela, J. Alcazar, F. Lauretani, R. Re, A. Botter, M. V. Franchi, I. Ara, S. Porcelli

**Affiliations:** 1 Department of Molecular Medicine, University of Pavia, Pavia, Italy; 2 GENUD Toledo Research Group, Faculty of Sport Sciences, University of Castilla-La Mancha, Toledo, Spain; 3 Centro de Investigación Biomédica en Red Fragilidad y Envejecimiento Saludable (CIBERFES), Instituto de Salud Carlos III, Madrid, Spain; 4 Department of Systems Biology, University of Alcalá, Madrid, Spain; 5 Faculty of Health Sciences, University of Castilla-La Mancha, Talavera dela Reina, Spain; 6 Geriatric Clinic Unit, Medical Geriatric Rehabilitative Department, University Hospital of Parma, Parma, Italy; 7 Department of Medicine and Surgery, University of Parma, Parma, Italy; 8 Department of Physics, Politecnico di Milano, Milano, Italy; 9 Institute for Photonics and Nanotechnologies, National Research Council, Milano, Italy; 10 Department of Electronics and Telecommunications, Laboratory for Engineering of the Neuromuscular System (LISiN), Politecnico di Torino, Torino, Italy; 11 Department of Biomedical Sciences, University of Padova (UNIPD), Padova, Italy; 12 Fondazione IRCCS Policlinico San Matteo, Pavia, Italy

**Keywords:** aging, maximal oxygen consumption, NIRS, running, wagner diagram

## Abstract

Aging is associated with declines in cardiorespiratory fitness and endurance performance, but this association is usually confounded by age-related declines in physical activity levels. For this reason, world-class master athletes serve as exceptional models for elucidating the limits of endurance performance in old age.

**Purpose:**

To examine the physiological responses to exercise and performance characteristics of an 81-year-old male runner who, in 2025, set a new world record in the 50-km race (4h47m39s, 10.5 km h^-1^) in the 80+ category.

**Methods:**

Two weeks after the world record, maximal O_2_ uptake ( $\dot{V}$ O_2max_), fractional utilization of $\dot{V}$ O_2max_ (lactate threshold [LT]), maximal fat oxidation (MFO) and running economy (RE) were assessed through incremental running tests. Limiting factors to $\dot{V}$ O_2peak_ were assessed during incremental cycling exercise by gas exchange, peak cardiac output (Q̇_peak_), and peak fractional O_2_ extraction of the vastus lateralis (VL) muscle. *In vivo* VL muscle oxidative capacity and relative resistance to O_2_ diffusion were estimated using near-infrared spectroscopy (NIRS) during repeated transient arterial occlusions in well-oxygenated (k_HIGH_) and low O_2_ availability (k_LOW_) conditions.

**Results:**

$\dot{V}$ O_2max_ was 52.8 mL kg^-1^·min^-1^, achieved at 13.2 km h^-1^. LT was attained at 10.5 km h^-1^. MFO was 0.55 g·min^-1^ occurring at 84% of $\dot{V}$ O_2max_ and RE was 237.5 mL kg^-1^·km^-1^. Cycling $\dot{V}$ O_2peak_ was 2.510 L min^-1^ (42.6 mL kg^-1^·min^-1^), Q̇_peak_ was 15.3 L min^-1^, and arterial-venous O_2_ difference was 16.4 mL dl^-1^, comparable to fractional O_2_ extraction around 75% obtained by NIRS. k_HIGH_ was 4.67 min^-1^ and k_LOW_ was 4.59 min^-1^, suggesting high oxidative and muscle O_2_ diffusing capacity.

**Conclusion:**

The exceptional endurance performance of this master athlete was attributed to his well-preserved $\dot{V}$ O_2max_ (to our knowledge the highest recorded in octogenarians, equivalent to the 70th percentile for healthy males aged 20–30 years) and a high fractional utilization of $\dot{V}$ O_2max_, together with a great ability to oxidize fats. Analyses on the limiting factors to $\dot{V}$ O_2max_ suggest that his exceptional performance was mostly due to the final steps of the oxygen cascade.

## Introduction

Endurance performance declines with age (Hawkins and Wiswell, 2003; Heath et al., 1981; Kaminsky et al., 2015; Lepers and Stapley, 2016; Tanaka and Seals, 2008; Valenzuela et al., 2020). Specifically, there is a curvilinear pattern of decline in peak endurance performance, with a modest decrease from the age of 35 to 50–60 years and more notable declines after the age of 60 (Tanaka and Seals, 2008). Among others, reductions in the maximal O_2_ uptake ( $\dot{V}$ O_2max_) appears to be one of the most important factors affecting endurance performance during aging, with a 5%–10% reduction per decade starting from the age of 30 (Hawkins and Wiswell, 2003; Heath et al., 1981; Kaminsky et al., 2015; Lepers and Stapley, 2016; Tanaka and Seals, 2008; Valenzuela et al., 2020).

$\dot{V}$ O_2max_ is considered the gold standard measurement of integrated cardio-pulmonary and muscle function, and it quantifies the maximal rate of adenosine triphosphate (ATP) regeneration required for sustained muscle contractions during endurance exercise. The generation of aerobic ATP is dependent on the delivery of O_2_ to muscle cells and its subsequent utilization via mitochondrial respiration. As elucidated by the conflation of Fick’s Principle and Fick’s Law of Diffusion, O_2_ flows from ambient air to the mitochondria by convection and diffusion, driven by pressure gradients against numerous resistances in series. The collective dynamics of these relationships establish the conceptual basis of the O_2_ cascade from lungs to the muscle tissue and enable the identification of specific limitations to $\dot{V}$ O_2max_. The age-related decline in $\dot{V}$ O_2max_ is mostly attributable to diminished cardiac output, consequent to a reduction in maximal heart rate and cardiac output (Tanaka et al., 2001), but a specific role can also be attributed to reductions in skeletal muscle mass and function (Fleg and Lakatta, 1988).

In the last few decades, the endurance performance of the world-class master athletes has improved more rapidly than that of their younger counterparts (Lepers and Stapley, 2016), despite the inevitable age-related performance decline (Tanaka and Seals, 2008). This trend is attributed to advances in training strategies, together with an increase in the number of master athletes competing in endurance events, but it is still debated what are the physiological determinants of the preserved endurance performance in some aged adults (Lanza et al., 2025; Marcinek and Ferrucci, 2025). Master athletes, defined as individuals older than ∼35 years who train and compete in organized competitive events, provide a unique model for studying how regular training can mitigate or delay age-related physiological decline, accounting for the confounding effect of reduced physical activity levels (Mckendry et al., 2018; Valenzuela et al., 2020). Notably, master athletes with an average age of 67 years can exhibit $\dot{V}$ O_2max_ values comparable to those of healthy adults 3 decades younger (Mckendry et al., 2018). This high exercise capacity lends further support to the hypothesis that a 5%–7% decline in $\dot{V}$ O_2max_ per decade is characteristic of master endurance athletes over the age of 45 (Pollock et al., 1997; Trappe et al., 2013).

In this case study, we examined the training characteristics, physiological profile and performance of a male endurance athlete who set the world record in a 50-km race in the 80+ age category in the 2025 Master Championship in Malaga (Spain). Moreover, to identify the O_2_ cascade profile, we tested the limiting factors of $\dot{V}$ O_2max_ using non-invasive measurements such as transthoracic bioimpedance and near-infrared spectroscopy (NIRS) during maximal cycling exercise.

## Methods

### Participant

An 81-year-old Spanish master athlete (height: 1.57 m; body mass: 58.9 kg; body mass index (BMI): 23.9 kg m^-2^) participated in this case study. With no prior training experience, he initiated running at the age of 66 and started competing at 70 years old in distances ranging from 800 m to 100-km ultramarathons, with a predominant focus on long-distance races. The athlete’s training was monitored during the last 12 months through the continuous recording of heart rate, training distance, and exercise intensity by a GPS-enabled heart rate monitor (Fenix 3, Garmin ltd., United States of America), previously validated (Carrier et al., 2020). This athlete is the current male marathon world champion in the 80+ age category (3 h, 39 min and 10 s in the 2024 Bucharest Marathon).

The participant volunteered to take part in the study after being informed about the procedures and potential risks. Written informed consent was obtained for each assessment session. The present study conformed to the standards set by the Declaration of Helsinki. The athlete was part of a larger project (TRAJECTOR-AGE) for examining longitudinal aging physiological decline (Lauretani et al., 2025). The study is registered at http://clinicaltrials.gov (NCT06168591) and was approved by the AVEN Ethical Committee (Emilia Romagna region, Italy) on 5 July 2022 (protocol #28022; study ID 283/2022/SPER/UNIPR).

#### Assessment overview

On 3 May 2025, the athlete set the world record at the Spanish 50-km Master Championship in Malaga, Spain, and we analyzed his performance during this race. Two weeks after he had achieved the 50 km world record, he attended our laboratory on 4 different occasions interspersed by at least 48 h. During the first testing session, he was interviewed about his entire sport career and training habits, hemoglobin concentration ([Hb]) was obtained from venous blood, and his body composition was assessed through dual-energy X-ray absorptiometry (DXA). On the second and third testing sessions, he performed a treadmill graded exercise test (GXT) and maximal fat oxidation (MFO) determination, respectively. On the last occasion, he performed several repetitions of moderate-intensity constant work-rate exercises (CWR), with repeated femoral artery occlusions at the end, and a cardiopulmonary cycling test (CPET).

#### Body composition assessment

The body composition analysis was performed using a DXA device (QDR Discovery Wi; Hologic, Bedford, MA, United States of America). All DXA scans were analyzed using Physician’s Viewer, APEX System Software Version 3.1.2. (Bedford, MA). Body composition parameters included lean mass (LM), fat mass (FM), bone mineral content (BMC) and bone mineral density (BMD) in the whole body. Regional analysis was conducted to evaluate FM and LM in upper- and lower-limbs and trunk. Daily quality control and calibration were performed with a phantom according to the manufacturer’s guidelines. Assessments were performed with the participant in a supine position, wearing light clothing free of metal, and without shoes or jewelry.

#### Blood sampling

Blood samples were collected after an overnight fast of at least 12 h with the participant in a seated position and in a resting condition. To determine [Hb], blood sample was drawn from an antecubital vein in 3 tubes containing ethylenediaminetetraacetic acid (EDTA) (BD Vacutainer, Stockholm, Sweden). Blood sample underwent assessment for routine clinical chemistry measurements.

#### Treadmill graded exercise test

The GXT was conducted during the athlete’s usual training hours, using his habitual competition footwear, having maintained his usual diet in the preceding days, avoided physical exercise for 24 h, and consumed no food in the 3 h prior to testing.

The GXT was performed on a treadmill (HP Cosmos Pulsar; H Cosmos Sports and Medical GMBH, Nussdorf Traunstein, Germany) with a slope of 1.0% to match the energy cost of running outdoors (Jones and Doust, 1996). The athlete performed a 5-min warm-up at 8 km h^-1^. The GXT started at the same velocity with 0.1 km h^-1^ increments every 10 s until exhaustion (the participant was secured through a safety harness).

Pulmonary ventilation ( $\dot{V}$ E, in BTPS [body temperature (37 °C), ambient pressure and gas saturated with water vapor]), oxygen consumption ( $\dot{V}$ O_2_), and carbon dioxide production ( $\dot{V}$ CO_2_), both in STPD (standard temperature [0 °C or 273 K] and pressure [760 mmHg] and dry [no water vapor]), were determined breath-by-breath by a metabolic cart (Quark CPET, Cosmed, Italy). Before each test, gas analyzers were calibrated with ambient air and a gas mixture of known concentration (O_2_: 16%, CO_2_: 4%) and the turbine flowmeter was calibrated with a 3-L syringe at three different flow rates. RER was calculated as $\dot{V}$ CO_2_/ $\dot{V}$ O_2_. HR was continuously recorded by chest band (HRM-Dual, Garmin ltd., United States of America).

$\dot{V}$ O_2max_ was defined as the highest $\dot{V}$ O_2_ averaged over a 30-s period and verified based on the following criteria (Petot et al., 2012): 1) $\dot{V}$ O_2_ increment <150 mL min^-1^; 2) maximal RER ≥1.10; 3) rating of perceived exertion (RPE) ≥ 15; 4) maximal heart rate (HR) > 85% of the age-predicted maximum.

Maximal aerobic speed (MAS) was defined as the velocity associated with the $\dot{V}$ O_2max_, while peak velocity (Vpeak) was the highest speed attained during the test (Lacour et al., 1991). Capillary blood lactate concentration (BLa) was assessed (Lactate Pro, Arkray, Japan) at rest and every 2 min during the GXT. The lactate threshold (LT) was defined as the highest sustainable speed that the participant could maintain while maintaining a BLa below 0.8 mmol L^-1^ increase from the resting value (Cerezuela-Espejo et al., 2018). RPE (Borg’s scale 6–20) (Borg, 1982) was recorded at the end of the test.

#### Maximal fat oxidation determination and running economy

The exercise testing protocol was adapted from validated protocols previously described (Jaén-Carrillo et al., 2025; Randell et al., 2017). The test was conducted on the same treadmill used for GXT, with a slope of 1.0% to replicate the energetic cost of outdoor running (Jones and Doust, 1996). The protocol began with a 6-min warm-up at an initial speed of 5.0 km h^-1^. From that point onward, speed was increased by 1 km h^-1^ every 4 min until a RER of 1.0 was reached.

Substrate oxidation was determined by means of average gas exchange measurements during the last 60 s of each 4-min stage of the incremental protocol (Amaro-Gahete et al., 2019a). Fat oxidation rate (g∙min^−1^) was calculated according to Frayn’s stoichiometric equations with the assumption that urinary nitrogen excretion was 0 g (Frayn, 1983). MFO rate and the speed at which MFO occurred (Fat_max_) were identified. Fat oxidation values were expressed in absolute terms (g·min^-1^), relative to body mass (mg·min^-1^·kg^-1^) and relative to lean mass (mg·min^-1^·kg^-1^). The relationship between fat oxidation rate and relative exercise intensity (%) was determined using a second-order polynomial curve, based on the fat oxidation value obtained at rest and all completed stages of the test. The polynomial curve was inspected by an experienced evaluator to ensure the best goodness-of-fit (i.e., *R* ^2^ > 0.70).

Running economy (RE) was derived from measurements of $\dot{V}$ O_2_ during the final minute of the 10 km h^-1^ submaximal stage, the closest to the average speed maintained by the athlete during the 50 km performance, and it was expressed as ml·kg^-1^·km^-1^. RER was <1.0, and $\dot{V}$ O_2_ steady state was confirmed visually and through regression based on two criteria: the absence of a significant slope in $\dot{V}$ O_2_ (P < 0.05) and a slope in $\dot{V}$ O_2_ of <150 mL min^-1^ (Robergs et al., 2010).

#### Muscle oxygen uptake recovery rate constant

The muscle oxygen uptake (m $\dot{V}$ O_2_) recovery rate constant (k) was measured using the approach recently proposed (Pilotto et al., 2022). With the participant seated on a cycle ergometer, oxygenation changes of the vastus lateralis were sampled at 10 Hz by a wireless, portable, continuous-wave, spatially resolved, NIRS device (Train.Red PLUS, Train.Red B.V., Netherlands), previously validated (da Mota Moreira et al., 2023). Briefly, this device is equipped with three fiber optic bundles: NIR light is emitted from three optodes at two wavelengths (760 and 850 nm) and received from a fourth optode for transmission back to the data acquisition unit to determine the relative concentrations of deoxygenated and oxygenated heme groups contained in hemoglobin (Hb) and myoglobin (Mb). This method does not distinguish between the contributions of Hb and Mb to the NIRS signal, but Mb signal was assumed to be of minor impact compared to the contribution of Hb (Grassi and Quaresima, 2016). Relative concentrations of deoxy-(hemoglobin + myoglobin) ([deoxy (Hb + Mb)]) and oxy-(hemoglobin + myoglobin) ([oxy (Hb + Mb)] were measured in the tissues ∼1.5–2 cm beneath the probe, with respect to an initial value obtained at rest before any procedure arbitrarily set equal to zero. From these measurements, relative changes in total hemoglobin and myoglobin ([tot (Hb + Mb)] = [oxy (Hb + Mb)] + [deoxy (Hb + Mb)]) and the Hb difference ([diff (Hb + Mb)] = [oxy (Hb + Mb)] – [deoxy (Hb + Mb)]) were calculated. In addition, the TSI (%) was measured using the spatially resolved spectroscopy approach (Ferrari et al., 2004). The skin at the NIRS probe site was shaved before the probe was placed longitudinally on the lower third of vastus lateralis muscle (∼10 cm above the knee joint), and secured with a black patch and elastic bandage. The thickness of the skin and subcutaneous tissue at the NIRS probe site (3.33 mm) was measured using an ultrasound device (MX7, Mindray, China). A 13 × 85-cm rapid-inflation pressure-cuff (SC12D; Hokanson, Bellevue, WA, United States of America) was placed proximally on the same thigh and attached to an electronically controlled rapid cuff-inflator (E20; Hokanson, Bellevue, WA, United States of America). After 2 min of rest, a prolonged arterial occlusion (300 mmHg) was performed until TSI plateaued. The cuff was instantly deflated and muscle reoxygenation was recorded until a steady-state was reached. This procedure was used to identify the physiological normalization (PN) of TSI which was standardized to 0% at the deflection point during the prolonged arterial occlusion (TSI min) and 100% at the maximum value reached during reperfusion (TSI max) (Adami et al., 2017). The 5-min cycling CWR was followed by an immediate stop and 10–20 intermittent arterial occlusions at 300 mmHg. Duration and timing of the repeated occlusions were controlled by the investigator to maintain TSI in two different ranges: from 0% to 10% of PN (LOW) and from 50% to 60% of PN (HIGH), where the total amplitude of PN was used as 0%–100% reference range. The HIGH range was selected to ensure that occlusions were performed under well oxygenated conditions, and to avoid a reduction in PO_2_ that could limit m $\dot{V}$ O_2_ (i.e., maintaining TSI above 50% of the physiological normalization) (Adami and Rossiter, 2018; Haseler et al., 2004). The LOW range was selected as the lowest boundary to evaluate m $\dot{V}$ O_2_ recovery k in poorly oxygenated conditions, without overstepping the deflection point (i.e., where TSI during occlusions loses linearity). The rate of muscle desaturation during each intermittent arterial occlusion (TSI, % s^−1^) was fitted to estimate the exponential m $\dot{V}$ O_2_ recovery or k, as described previously (Adami et al., 2017). Data were quality checked before curve fitting to remove invalid values or outliers, i.e., low initial TSI values, or incomplete occlusions (Beever et al., 2020). Subsequently, the difference between these conditions was calculated (Δk = k_HIGH_–k_LOW_).

#### Cycling cardiopulmonary exercise test

The CPET consisted of an incremental ramp test on the cycle ergometer. Power output was increased 20 W every minute starting from the initial unloading condition. The participant was instructed to maintain constant cadence at his preferred value (∼70 rpm). Intolerance was defined when the participant could no longer maintain his chosen pedaling frequency despite verbal encouragement.

$\dot{V}$ E, $\dot{V}$ O_2_, and $\dot{V}$ CO_2_ were determined breath-by-breath by a metabolic cart (Quark CPET, Cosmed, Italy) as described in the ‘Treadmill graded exercise test’ section). RER was calculated as $\dot{V}$ CO_2_/ $\dot{V}$ O_2_ and HR was recorded by using 12-lead ECG (ECG 12X, Cosmed, Italy). RPE (Borg® 6–20 (Borg, 1982)) was determined at the end of the test. Peak cardiopulmonary variables were measured from the highest 30 s mean values prior to intolerance when at least two of the following criteria were found: 1) $\dot{V}$ O_2_ increment <150 mL min^-1^; 2) maximal RER ≥1.10; 3) rating of perceived exertion (RPE) ≥ 15; 4) maximal heart rate (HR) > 85% of the age-predicted maximum.

Stroke volume (SV) was estimated beat-by-beat by means of transthoracic bioimpedance cardiography (PhysioFlow, Manatec Biomedical, France) and averaged every 10 beats. The accuracy of this device has been previously evaluated during incremental exercise in healthy subjects against the direct Fick method (Richard et al., 2001). A detailed description of the method has been provided elsewhere (Charloux et al., 2000). HR was obtained from the R-R interval determined on the ECG first lead. Cardiac output (Q̇) was then calculated by multiplying SV and HR.

Oxygenation changes in the vastus lateralis muscle were evaluated continuously during the tests by NIRS (Ferrari et al., 2004). As in previous studies (see, e.g., (DeLorey et al., 2003; Ferreira et al., 2007; Ferri et al., 2007; Grassi et al., 2003; Grassi et al., 2007; Kowalchuk et al., 2002; Lanfranconi et al., 2006; Porcelli et al., 2010)) [deoxy (Hb + Mb)] was taken as an estimate of skeletal muscle fractional O_2_ extraction, because this variable, unlike [oxy (Hb + Mb)], is relatively insensitive to changes in blood volume (Ferrari et al., 1997; Grassi and Quaresima, 2016). Because these data are expressed in arbitrary units, a prolonged arterial occlusion was used as physiological calibration and data obtained during the exercise protocol were expressed as a percentage of the values determined by the maximal deoxygenation of the muscle, i.e., Δ[deoxy (Hb + Mb)] plateau during ischemia. For more technical details of the measurement, see Porcelli et al. (2012).

## Results

### 50-km race performance

**FIGURE 1:**
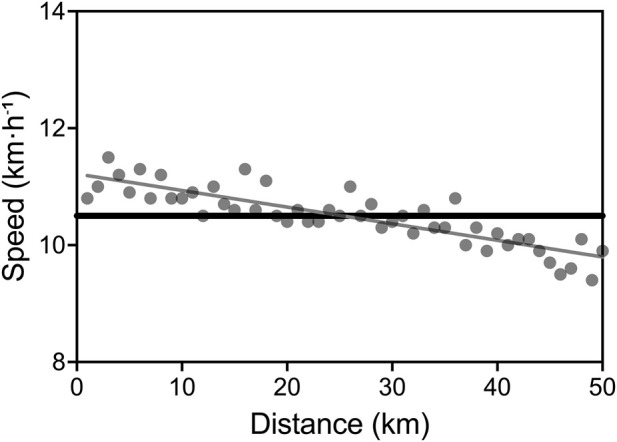
Pacing during the Spanish 50-km Master Championship. Grey circles represent the actual speed for each kilometer. Black line identifies the 50-km average speed.

The athlete completed the 50-km distance in 4 h, 47 min, and 39 s, corresponding to an average speed of 10.5 km h^-1^ (5:44 min km^-1^). With this performance, he broke the men’s 50-km world record in the 80+ age category, improving the previous record by 49 min and 2 s. The former record had been held by Josef Mathias Simon of Luxembourg since 2015, with a time of 5:36:41. The slowest kilometer was completed in 6 min and 21 s (corresponding to 9.4 km h^-1^), whereas the fastest kilometer was run at a pace of 5:18 min km^-1^, corresponding to an average speed of 11.3 km h^-1^ (Figure 1).

#### Training characteristics

The athlete reported having performed regular endurance exercise for more than 10 years. His training program followed a linear periodization model. He ran between 65 km per week in general non-competitive phases, while this volume increased up to 120 km per week in specific phases (i.e., in the ∼2 months preceding the target competition). Weekly frequency ranged from 6 to 7 sessions. Annually, the subject covered over 3,500 km.

Exercise sessions were divided into two main types: continuous training and interval training. During the general phases, the subject exclusively performed continuous training, typically at a pace ranging from 5:00 to 6:00 min km^-1^. The specific phase also included interval training, which initially started with 200-m intervals and progressively increased in distance throughout the training period (e.g., 400 m, 800 m, 1 km, 2 km, 4 km), reaching intervals of up to 8 km. These intervals were usually performed at a pace 5–10 s km^-1^ faster than the intended race pace.

#### Body composition

The subject’s anthropometric measurements were as follows: height 1.57 m, body mass 58.9 kg, and BMI 23.9 kg/m^2^. DXA analysis revealed a FM of 11.65 kg (19.5%), LM of 46.00 kg (76.8%), BMC of 2.24 kg, and BMD of 1.14 g/cm^2^ in the whole body. Regional analyses showed FM of 3.57 kg (18.9%) and LM of 14.45 kg in the lower limbs. In the upper limbs, FM totaled 1.34 kg (20.0%) while LM reached 5.01 kg. The trunk region contained 5.77 kg (19.1%) of FM and 23.86 kg of LM.

#### $\dot{V}$ O_2max_ and metabolic thresholds

**FIGURE 2:**
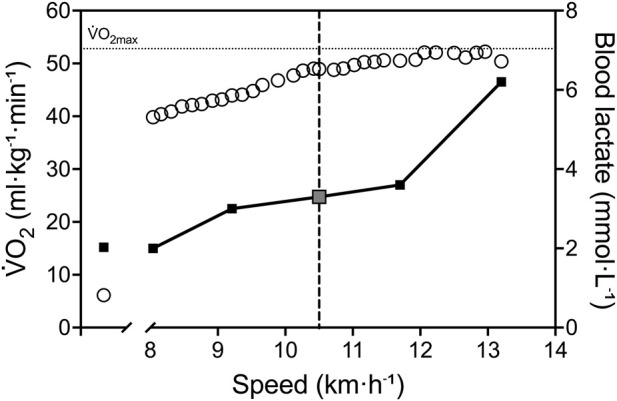
Physiological response during treadmill GXT. White circles represent $\dot{V}$ O_2_ and black squares represent BLa. The vertical dashed line identifies speed of the 50-km world record (10.5 km h^-1^), interestingly corresponding to LT (grey square). The horizontal dotted lines represent the highest 30 s $\dot{V}$ O_2_ average. $\dot{V}$ O_2_, oxygen consumption; BLa, blood lactate concentration; LT, lactate threshold.

From treadmill GXT, the participant showed a $\dot{V}$ O_2max_ of 52.8 mL kg^-1^·min^-1^. Vpeak was 13.2 km h^-1^. He reached a maximal HR of 155 bpm, and peak BLa of 6.2 mmol L^-1^. His final RPE was 19. LT occurred at 10.5 km h^-1^ (80% of Vpeak), corresponding to a $\dot{V}$ O_2_ of 48.3 mL kg^-1^·min^-1^ (91% of $\dot{V}$ O_2max_) (Figure 2).

#### Maximal fat oxidation determination and running economy

**FIGURE 3:**
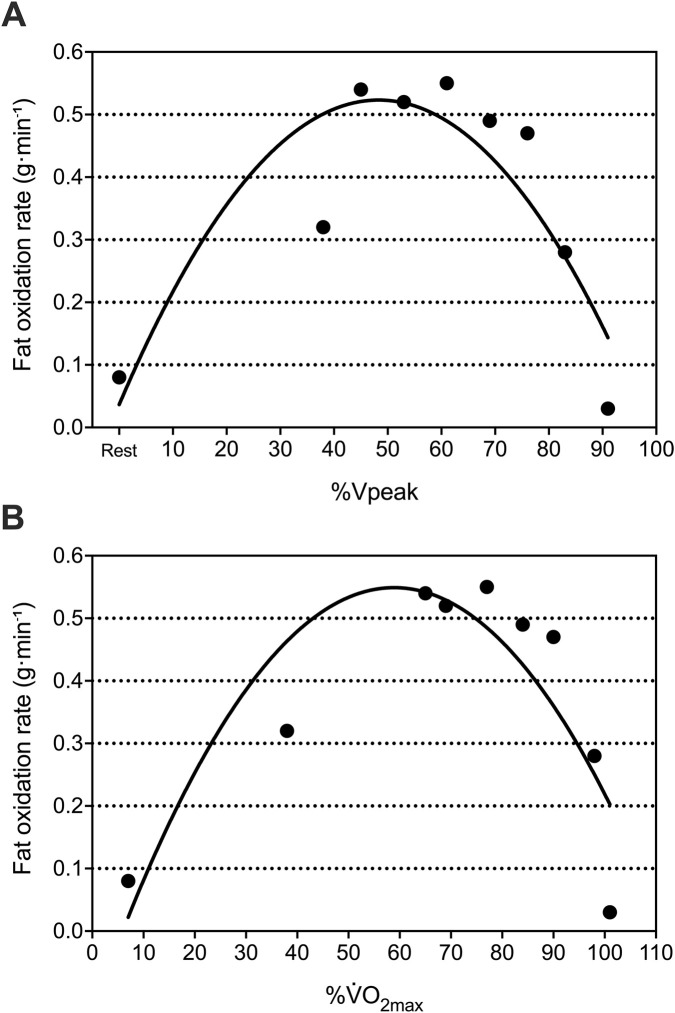
Relationship between fat oxidation rate and relative exercise intensity (%) expressed as Vpeak panel **(A)** and $\dot{V}$ O_2max_ panel **(B)** determined using a second-order polynomial curve, based on the fat oxidation value obtained at rest and all completed 4-min stages of the step incremental protocol. Vpeak, peak velocity achieved during treadmill graded exercise test; $\dot{V}$ O_2max_, maximal oxygen consumption achieved during treadmill graded exercise test.

His absolute fat oxidation rate at rest was 0.08 g min^-1^, equivalent to 1.36 mg min^-1^·kg^-1^ relative to body mass and 1.74 mg min^-1^·kg^-1^ relative to lean mass. His absolute MFO was 0.55 g min^-1^, corresponding to 9.34 mg min^-1^·kg^-1^ relative to body mass and 11.96 mg min^-1^·kg^-1^ relative to lean mass (Figure 3). MFO occurred at a velocity of 8 km h^-1^, equivalent to 61% of Vpeak and 77% of $\dot{V}$ O_2max_. $\dot{V}$ O_2_ at 10 km h^-1^ was 46.4 mL kg^-1^·min^-1^, corresponding to a RE of 237.5 mL kg^-1^·km^-1^.

#### Muscle oxygen uptake recovery rate constant

Muscle oxidative capacity was characterized by a recovery rate constant k in HIGH range of 4.67 min^-1^ k_LOW_ was 4.59 min^-1^, resulting in Δk value of 0.07.

#### CPET

**FIGURE 4:**
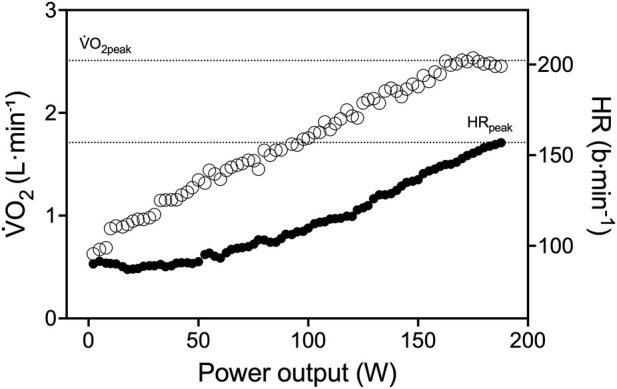
Physiological response during maximal cycling CPET. White circles represent $\dot{V}$ O_2_, and black circles represent HR. The dotted lines represent the highest 30 s $\dot{V}$ O_2_ and HR average. $\dot{V}$ O_2_, oxygen consumption; HR, heart rate.

The cycling CPET revealed a $\dot{V}$ O_2peak_ of 42.6 mL kg^-1^·min^-1^ (2.510 L min^−1^) attained at a peak power output of 189 W. At exhaustion, VE was 95.1 L min^-1^ and RER was 1.12 (Figure 4). His final RPE was 16. HR was 156 bpm (112% of age-predicted), stroke volume was 98 mL, and cardiac output was 15.3 L min^-1^. Calculated CaO_2_ was 214.4 mL L^-1^ and maximal oxygen delivery (Q̇aO_2_) was 3.281 L min^-1^, using the formula [CaO_2_ = (1.36 × [Hb] × SaO_2_) + (0.003 × PaO_2_)], the [Hb] of 16.1 g dl^-1^ and assuming at the end of exercise an arterial saturation (SaO_2_) of 98%, arterial partial pressure of O_2_ (PaO_2_) of 100 mmHg and muscle pH and temperature 7.4 °C and 37 °C, respectively. From Fick’s equation, Ca-vO_2_ resulted 164.1 mL L^-1^, corresponding to an O_2_ extraction of 76.5%, similar to the fractional O_2_ extraction (Δ[deoxy (Hb + Mb)]_peak_/Δ[deoxy (Hb+Mb)_ischemia_] = 75.2%) estimated by NIRS.

Whole-body oxygen diffusion capacity (DO_2_) revealed a value of 75.3 mL min^-1^·mmHg^-1^, assuming mitochondrial partial pressure is very low pressure during maximal exercise and so could be neglected (Richardson et al., 2001).

## Discussion

In this case study, we analyzed the performance and the physiological profile of an 81-year-old Spanish athlete who broke the 50-km world record for men over 80 years of age in May 2025, and currently holds first place in the marathon world championship in the 80+ age category (2024-2025). The superior endurance performance observed in this master athlete was primarily explained by a well-preserved $\dot{V}$ O_2max_, combined with a high fractional utilization of $\dot{V}$ O_2max_ and an enhanced capacity for fat oxidation. When limiting factors to $\dot{V}$ O_2max_ were explored on the cycle-ergometer, we observed fairly normal age-related cardiac output but highly preserved muscle oxidative and diffusive capacity. The unique data collected in our octogenarian elite athlete illustrate how endurance training in the late phase of life can attenuate or delay physiological changes associated to aging, thereby contributing to the characterization of healthy aging phenotypes.

Although age-related performance decline is inevitable (Tanaka and Seals, 2008), largely due to reductions in $\dot{V}$ O_2max_ associated with diminished cardiac output (Tanaka et al., 2001) and decrease in skeletal muscle mass and function (Fleg and Lakatta, 1988), regular physical activity may serve as an effective countermeasure (Valenzuela et al., 2020), eliciting beneficial adaptations at both the cardiovascular level and within skeletal muscle. Indeed, structural, functional, and electrical cardiac remodeling resulting from the physical and metabolic load placed on the heart (Beaudry et al., 2016) as well as improvements in muscle mass and mitochondrial capacity follow exercise training (Grevendonk et al., 2021).

In this study we collected functional indexes of endurance performance in an octogenarian elite athlete. The incremental running test showed a very high cardiorespiratory fitness relative to his age, as indicated by a $\dot{V}$ O_2max_ of 52.8 mL kg^-1^·min^-1^, which is to the best of our knowledge the highest value reported for an individual older than 80 years (the previous value was 50 mL kg^-1^·min^-1^) (Karlsen et al., 2015). For comparison, untrained age-matched individuals present values ranging from 18 to 25 mL kg^-1^·min^-1^ (Trappe et al., 2013), and the $\dot{V}$ O_2max_ obtained is equivalent to the 70th percentile for healthy males in their 20–30s (Liguori et al., 2022). As expected, this value was 19% lower when the athlete exercised on the cycle ergometer (2.510 vs. 3.110 L min^-1^ in cycling and running, respectively) in accordance with the smaller muscle mass involved in the activity and previous studies showing lower $\dot{V}$ O_2max_ values in cycling compared to running test (14%–18% range) on running athletes (Bouckaert et al., 1990; Fernhall and Kohrt, 1990; Moreira-da-Costa et al., 1989). However, cycling $\dot{V}$ O_2max_ was still significantly higher than the values reported for untrained subjects of the same age (Trappe et al., 2013). This might also have clinical implications, particularly given that a higher $\dot{V}$ O_2max_ has been linked to a lower mortality risk even at the most advanced ages (Kokkinos et al., 2022).

To test the central limiting factors of $\dot{V}$ O_2max_, heart rate, stroke volume, and cardiac output were monitored during the maximal cycling exercise. The maximal heart rate was lower compared to that of young athletes (∼190 bpm at 25 years old), although higher than predicted for his age (112%) (Tanaka et al., 2001). This result is not surprising if we consider ageing-related adaptations in autonomic control of heart rate (Tanaka et al., 2001). The stroke volume (128 mL) was also lower compared to young individuals (Zhou et al., 2001), in line with the decrease in cardiac tissue stiffness with age (Lakatta and Levy, 2003). The combination of these two factors resulted in a maximal cardiac output of 15.3 L min^-1^ (corresponding to 9.6 L min^-1^·m^-2^ when normalized for body surface area), a value comparable to those recorded by cardiac blood pool imaging and echocardiography technique in 65-year-old untrained males subjects (16.7 L min^-1^) but lower than the one of trained master athletes (19.1 L min^-1^) (Seals et al., 1994). Other authors found higher peak exercise stroke volume (200 mL) and cardiac output (22.2 L min^-1^, 11.4 L min^-1^·m^-2^ normalized for body surface area) in the former marathon world-record holder aged 77 years old (Foulkes et al., 2024). The difference in cardiac function between our athlete and the one recruited by others in the previous report may be due to the transient interruption of exercise training that our athlete faced in the middle aged. Indeed, detraining may have affected cardiac stiffness and blood/plasma volume, limiting ventricular filling (Carrick-Ranson et al., 2023; Coyle et al., 1986; Foulkes et al., 2024).

However, in the present athlete the reduction found in cardiac function was well compensated by a high value of Hb concentration, which allows for a large maximal oxygen delivery (Q̇aO_2_) (3.281 L min^-1^), higher than age-matched untrained individuals (∼2.900 mL L^-1^, (Capelli et al., 2025)).

Nevertheless, the unique endurance performance and the high values of $\dot{V}$ O_2max_ were associated with remarkable peripheral adaptations at the level of the skeletal muscle. Indeed, the arterial-venous O_2_ difference calculated was 164 mL L^-1^, corresponding to an O_2_ extraction of 75%, and the muscle oxidative capacity, as estimated by the muscle $\dot{V}$ O_2_ recovery rate constant (4.67 min^-1^) by NIRS was even better than young endurance-trained individuals (Brizendine et al., 2013). Classically, aging is associated with a progressive decline in skeletal muscle mitochondrial content and function, contributing to metabolic dysfunction in older adults (Fleg and Lakatta, 1988). However, exercise training can largely negate these age-related effects, as trained older adults exhibit higher levels of oxidative phosphorylation proteins and a preserved mitochondrial network. Although this topic is still debated in literature (Lanza et al., 2025; Marcinek and Ferrucci, 2025), our results seem to support the thesis that preserved exercise training habits have beneficial effects on mitochondrial capacity in aging populations (Grevendonk et al., 2021; Hood et al., 2019).

**FIGURE 5:**
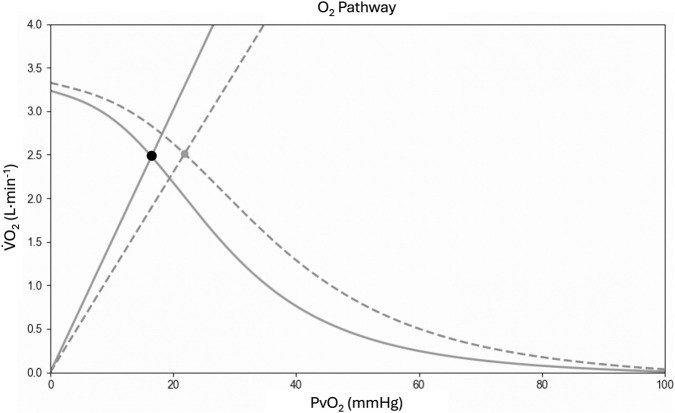
Wagner diagram based on cardiopulmonary cycling test data. In the graph, diagram from the present athlete (solid line) is compared with young healthy subjects from Mandic et al. (Goulding, 2024; Mandić et al., 2023) (dashed line). Fick’s principle (curved line) is conflated with Fick’s law of diffusion (straight line), with the point of intersection yielding the measured maximal oxygen uptake ( $\dot{V}$ O_2max_; black circle). The slope of the straight line gives the diffusing capacity for O_2_ (DO_2_) of the muscle. The point at which the curved line denoting Fick’s principle crosses the ordinate yields the maximal convective O_2_ delivery (Q̇aO_2_), i.e., the maximal cardiac output (Q̇) multiplied by arterial O_2_ content (CaO_2_). The point where the curve crosses the abscissa is the theoretical $\dot{V}$ O_2_ of zero where no O_2_ extraction occurs, hence venous PO_2_ (PvO_2_) is equal to arterial PO_2_ (PaO_2_). The figure was produced using the Helsinki O_2_ Pathway Tool (Rissanen et al., 2024).

To better understand physiological adaptations in the O_2_ cascade from the lungs to the mitochondria, we utilized the collected parameters to reconstruct the Wagner diagram (Figure 5) through the Helsinki O_2_ Pathway Tool (Rissanen et al., 2024). This approach has been recently used by Goulding to demonstrate the role muscle diffusive capacity in response to sprint interval training from data collected by Mandic et al. (Goulding, 2024; Mandić et al., 2023). In Figure 5 it is possible to appreciate the unique features of our athlete in comparison to young healthy subjects. Although the $\dot{V}$ O_2max_ was similar between our athletes and young population, it is interesting to note that the calculated value of whole-body diffusion capacity corresponded to 75.3 mL min^-1^·mmHg^-1^, demonstrating outstanding diffusion capacity, higher than young healthy subjects (i.e., range 55–70 mL min^-1^·mmHg^-1^, (Goulding, 2024; Mandić et al., 2023)). This result was supported by the Δk value close to zero (0.07 min^-1^). Δk is a non-invasive approach applied at the level of the skeletal muscle that utilizes NIRS data to collect information about relative muscle O_2_ diffusion resistance. More specifically, the m $\dot{V}$ O_2_ recovery rate constant is measured in non-limiting (i.e., HIGH tissue saturation index ranges) and limiting O_2_ availability (LOW conditions), and the change in the recovery rate is related to limitations to intramuscular O_2_ flux (Pilotto et al., 2022; Villanova et al., 2025). Thus, our athlete showed large adaptations in both oxidative and O_2_ diffusion capacity in the skeletal muscle. These data support recent calculations made on $\dot{V}$ O_2_max values in subjects ranging from 30 to 85–90 years old where authors reported a progressive decrease in $\dot{V}$ O_2_ with aging associated to relevant impairments in peripheral resistance to O_2_ muscular utilization rather than to reductions in the maximal cardiovascular transport of oxygen (Capelli et al., 2025). In this work, there was no specific information about the training status of the subjects but in our athlete we can speculate the observed peripheral adaptations were determined by a large volume endurance training, together with some high-intensity interval training sessions, which can positively affect muscle mass, capillary adaptations, and mitochondrial function/content (Liu et al., 2022; Bishop et al., 2014).

The exceptional physiological characteristics observed during exhaustion were also accompanied by unique submaximal features. The lactate threshold occurred at a velocity of 10.5 km h^-1^ (80% of Vpeak) and corresponded to 91% of $\dot{V}$ O_2max_. Such elevated levels of relative intensity are consistent with the physiological profiles of elite master athletes over 70 years of age, where lactate thresholds approaching 93% of $\dot{V}$ O_2max_ have been observed (Robinson et al., 2019). This value was higher than that observed in trained, but not elite, male athletes over the age of 60 and 70, where the lactate threshold (LT) corresponded respectively to 76.8% and 73.5% of $\dot{V}$ O_2max_ (Wiswell et al., 2000). The relative intensity of LT is also significantly higher than the one observed in young trained athletes (26 years old, $\dot{V}$ O_2max_ 60.2 mL kg^-1^·min^-1^), which showed a velocity at LT of 65.2% of maximal aerobic speed (Cerezuela-Espejo et al., 2018), and in elderly untrained (72 years old), where LT corresponded to ∼60% of $\dot{V}$ O_2max_ (Takeshima et al., 1996). The elevated LT intensity observed in this athlete may be explained by his specific training regimen, which predominantly consists of high-volume exercise performed near LT intensity, with no inclusion of sessions targeting $\dot{V}$ O_2max_ intensities.

MFO corresponding to 0.55 g min^-1^ in this master athlete is comparable to normative data found in young athletic population (0.60 g min^-1^ for men) (Achten et al., 2003; Amaro-Gahete et al., 2019b). Furthermore, his Fat_max_ was found at 77% of $\dot{V}$ O_2max_, a value considerably higher compared to younger individuals (∼50%) (Achten et al., 2003; Amaro-Gahete et al., 2019b). The pronounced fat oxidation capacity observed may be attributed to the elevated muscle oxidative capacity, reflecting mitochondrial adaptations in both function and content that are closely associated with enhanced mitochondrial fatty acid oxidation (Dandanell et al., 2018), or alternatively reflects metabolic remodeling associated with keto-adaptation (Noakes et al., 2023). The record-holder peripheral advantages can be attributed to his specific training characteristics.

Among the physiological factors influencing running performance, we also evaluated O_2_ cost of exercise. The athlete’s running economy was lower than the typical values reported for elite younger males (39.9 mL kg^-1^·min^-1^ at 14 km h^-1^) and to those observed in younger recreational runners (36.7 mL kg^-1^·min^-1^ at 10 km·h^-1^) (Barnes and Kilding, 2015). This value contrasts the exemplary running economy value (179 mL kg^-1^·km^-1^ at 12 km h^-1^) demonstrated by a 70-year-old male marathon world record holder master athlete (Van Hooren and Lepers, 2023). We do not have a clear hypothesis for these differences, but it should be noted that our athlete is 10-year older than the marathon world record holder, and this gap may have affected tendon stiffness (Karamanidis and Arampatzis, 2006; Mademli and Arampatzis, 2008), resulting in a significant negative impact on running economy. Moreover, our athlete reported an average running distance of 65 km wk^-1^, with a maximum of 120 km wk^-1^ in the specific phases. In comparison, the 70-year-old male master marathon world record holder routinely ran 135–140 km wk^-1^ (Van Hooren and Lepers, 2023). Thus, the lower weekly distance could have led to smaller adaptations that enhanced running economy (Morgan et al., 1995). It is also important to note that the O_2_ cost of running was measured in fresh condition while it would have been of interest to have information about the running economy along the different segments of the 50-km race to better describe the unique performance (Zanini et al., 2024; Scheer et al., 2018). However, the race monitoring data revealed a 10% decrease in running speed, which is more pronounced than the 5% decrease observed in 40-year-old ultradistance running athletes following a 60-km ultramarathon (Schena et al., 2014).

## Limitations

This study captures an informative snapshot of the athlete’s physiology close to the 50-km record. More specifically, the distance from the establishment of the new 50-km record and the tests was 2 weeks. This is the optimal timeframe for evaluating physiological characteristics in proximity to performance, while simultaneously avoiding the inclusion of detrimental acute effects associated with long-distance running. Thus, our results seem to support an outstanding physiological profile of our athlete.

Nevertheless, it should be considered that we did not have longitudinal data that allowed us to trace the developmental trajectory or identify factors that shaped the physiological adaptations underlying this performance. Moreover, in the present study we investigated the physiological determinants of $\dot{V}$ O_2max_ by dissecting some of the steps along the O_2_ cascade and linked their specific adaptations to training. However, we cannot exclude that the present athlete had unique genomic markers related to endurance performance (Psatha et al., 2024). Thus, future studies should try to follow longitudinal approaches and focus their attention on the link between outstanding athletic performance and unique genetic profiling.

Additionally, the comparisons of physiological determinants between our athlete and data from the literature was used to help the readers in better understanding the excellence of this case report. This approach was strengthen using comparable experimental approach between the present study and previous literature (Cerezuela-Espejo et al., 2018; Jaén-Carrillo et al., 2025; Randell et al., 2017), as well as high reproducible and valid testing. However, there are differences between exercising in the laboratory setting and performing a race on the field. For example, running economy was evaluated on a 1% uphill treadmill slope to more accurately reflect the energy cost of outdoor running (Jones and Doust, 1996) but there is likely a significant difference in running economy when running on a trail vs. running the same speed on a treadmill (Sabater et al., 2023). Thus, future study should try to explore physiological limitations of performance by also collecting data during actual athletic performance.

Finally, the approach used in the present study to identify limiting factors of $\dot{V}$ O_2max_ and the O_2_ cascade profile involved a thorough examination of each factor mainly based on non-invasive techniques and several assumptions regarding blood composition (SaO_2_, PaO_2_, pH and temperature) during maximal exercise. Furthermore, the Helsinki O_2_ Pathway Tool was used to calculate whole-body oxygen delivery and diffusion capacity, which allowed an estimation of the average systemic capacity to move and diffuse oxygen from blood vessels to cell, but not the specific response of the lower limbs. The readers should be aware that these approaches have some limitations compared to the direct Fick’s approach or invasive cardiopulmonary testing. For example, in this study arterial O_2_ delivery to the muscles was estimated using Hb concentration in the venous blood but several conditions, and especially endurance training, lead to plasma volume expansion and can result in hemodilution, manifesting as reduced hematocrit and hemoglobin concentration (Montero and Lundby, 2018). However, the high degree of consistency between whole-body measurements and specific vastus lateralis variables (i.e., muscle oxygen diffusion capacity, as well as similarity in arterio-venous O_2_ extraction calculated using Fick’s Principles or NIRS-derived Δ[deoxy (Hb + Mb)]) support the physiological meaning of the present results.

## Conclusion

This case report on the world-record holder for the 50-km running distance in the male 80+ age category revealed the highest $\dot{V}$ O_2max_ recorded so far in octogenarians. Although running economy is lower than elite athletes and comparable to those of recreational runners, his performance is driven by relatively high fat oxidation metabolism and exceptional oxygen fractional utilization. The latter is explained by extraordinary skeletal muscle adaptation such as high oxidative and diffusive capacity, demonstrating well-adapted factors at the last steps of the oxygen cascade. Thus, the present findings reinforce the concept that maintaining high exercise capacity in advanced age supports the preservation of $\dot{V}$ O_2max_, a key predictor of all-cause mortality, drawing attention to specific adaptations at the level of the skeletal muscle.

## Data Availability

The raw data supporting the conclusions of this article will be made available by the authors, without undue reservation.
